# Multiwalled carbon nanotube based aromatic volatile organic compound sensor: sensitivity enhancement through 1-hexadecanethiol functionalisation

**DOI:** 10.3762/bjnano.10.227

**Published:** 2019-12-04

**Authors:** Nadra Bohli, Meryem Belkilani, Juan Casanova-Chafer, Eduard Llobet, Adnane Abdelghani

**Affiliations:** 1 Carthage University, National Institute of Applied Science and Technology, Research Unit of Nanobiotechnology and Valorisation of Medicinal Plants UR17ES22, Bp 676, Centre Urbain Nord, 1080 Charguia Cedex, Tunisia; 2 Tunis University, ENSIT, Avenue Taha Hussein, Montfleury, 1008 Tunis, Tunisia; 3 MINOS-EMaS, Universitat Rovira i Virgili, Avda. Països Catalans, 26, 43007 Tarragona, Spain

**Keywords:** gold-decorated MWCNTs, multiwall carbon nanotubes (MWCNTs), self-assembled monolayers (SAMs), sensitivity, selectivity, vapour sensor

## Abstract

Aromatic volatile organic compound (VOC) sensors are attracting growing interest as a response to the pressing market need for sensitive, fast response, low power consumption and stable sensors. Benzene and toluene detection is subject to several potential applications such as air monitoring in chemical industries or even biosensing of human breath. In this work, we report the fabrication of a room temperature toluene and benzene sensor based on multiwall carbon nanotubes (MWCNTs) decorated with gold nanoparticles and functionalised with a long-chain thiol self-assembled monolayer, 1-hexadecanethiol (HDT). High-resolution transmission electron microscopy (HRTEM) and Fourier transform infrared spectroscopy (FTIR) were performed to characterize the gold nanoparticle decoration and to examine the thiol monolayer bonding to the MWCNTs. The detection of aromatic vapours using Au-MWCNT and HDT/Au-MWCNT sensors down to the ppm range shows that the presence of the self-assembled layer increases the sensitivity (up to 17 times), selectivity and improves the response dynamics of the sensors.

## Introduction

Aromatic volatile organic compounds (VOCs) such as benzene and toluene are hazardous vapours causing considerable damage to human health upon extended exposure. Benzene, for example, is known to have a carcinogenic effect on exposed humans. Since 2008, toluene and benzene monitoring is mandatory by the European Air Quality Directive, where the upper and lower assessment thresholds for benzene are limited to 0.6 ppb and 1.05 ppb, respectively. The monitoring method required by the European directive is complex, expensive and time consuming. It is carried out through active/online sampling, using desorption and gas chromatography, which are hardly portable or practical methods for implementing widespread, continuous indoor monitoring [1]. These facts have prompted the scientific community to work intensively on the development of cost effective, sensitive and reliable sensors for environmental pollution monitoring.

Furthermore, besides the prevention of indoor exposure to harmful aromatic VOCs, a new application has arisen recently for the development of such gas sensors. In fact, recent scientific evidence has shown a correlation between the presence of some VOCs in exhaled human breath and the presence of disease. For instance, the presence of trace concentrations of toluene in exhaled breath is associated with lung cancer and can therefore be considered as a biomarker for this pathology [2–4].

A gas sensor is generally composed of an active sensing film or material deposited on an electrode. The sensing performance is strongly correlated to the active sensing film/material used. Various nanomaterial-based gas sensors have been investigated to monitor the presence of aromatic VOCs. The ones mainly studied are based on metal oxides, carbon nanotubes, graphene and hybrid materials [5–6].

Carbon nanotube based gas sensors (e.g., single-wall carbon nanotube (SWCNT), multiwall carbon nanotube (MWCNT), graphene, graphene oxide (GO)) present a sensitive active layer exhibiting an electrical resistance change while in contact with the target gas due to interactions at the molecular level [7–8]. These interactions, depending whether they are strong covalent (chemisorption) or weak (physisorption), highly impact the sensor performance, that is, the sensitivity, response and recovery time, and detection range. Unlike metal-oxide-based gas sensors, CNT-based sensors operate at room temperature (low activation energy) and can therefore lead to the development of commercially affordable sensors [9–11]. However, they suffer from some limitations such as their poor selectivity, partial recovery and long response recovery time [12]. To overcome these issues, several strategies have been reported including, but not limited to, metal decoration or chemical functionalisation [13].

In the present work, we investigated the effect of gold nanoparticle decorated, multiwall carbon nanotubes functionalized with 1-hexadecanethiol on the sensor selectivity and sensitivity towards benzene and toluene vapours.

## Experimental

### Materials

Multiwall carbon nanotubes (MWCNTs) were purchased from Nanocyl S.A. (Belgium) with a minimum purity of 95 wt %. They have an average length of 50 µm and average inner/outer diameter of 3 and 15 nm, respectively. 1-Hexadecanethiol (HDT) was purchased from Sigma-Aldrich.

### Sensor fabrication

#### Gold nanoparticle decoration of MWCNTs

Prior to their deposition on the interdigitated electrode surface, the MWCNTs were treated by oxygen plasma to create oxygen vacancies on the walls of the CNTs in order to enhance their surface reactivity [14–15]. The detailed description of the experimental steps undertaken is presented in Supporting Information File 1 [16–18]. The MWCNTs were then dispersed in dimethylformamide (DMF) (0.1 mg MWCNTs in 1 mL of DMF) using an ultrasonic bath for 20 minutes at room temperature. Then, they were air-brush deposited over the platinum interdigitated electrode area of alumina substrates. They were then decorated with gold nanoparticles via a sputtering technique with a Sputtering ATC Orion 8-HV-AJA International machine [19]. This technique consists of bombarding the surface of a gold disc by a plasma beam, enabling the nanoparticles to cling to the walls of the MWCNTs under the effect of nucleation. The power and time required for the plasma beam aperture within the cell were optimized and fixed to 30 W and 10 seconds, respectively.

#### Functionalisation with self-assembled monolayers (SAMs)

**Figure 1:**
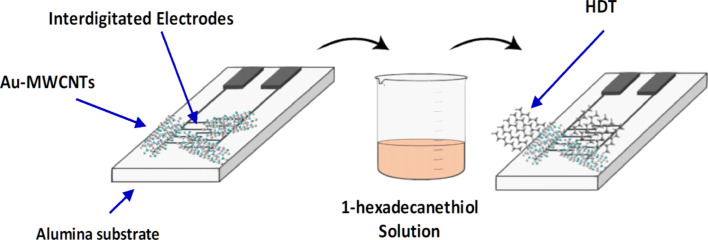
Synoptic structure of the sensor before and after the HDT deposition.

The Au-decorated MWCNT substrate was further functionalized with an alkanethiol self-assembled monolayer of 1-hexadecanethiol (HDT). The sensor functionalisation was undertaken through its immersion for 4 hours at room temperature in a solution of 5 mM of HDT diluted in ethanol. The sensor was then rinsed with ethanol to wash off the unbound thiol molecules and dried under a nitrogen stream [20]. Figure 1 shows the synoptic structure of the sensor before and after the HDT deposition.

#### HRTEM and FTIR characterisation

The analysis of the quantity and distribution of the gold nanoparticles attached to the MWCNTs was undertaken with a high-resolution transmission electron microscope (JEOL 1011), operating at 100 kV. An Alpha FTIR spectrometer (Bruker, France) equipped with an ATR platinum crystal diamond module was used, in absorbance mode, to obtain the infrared spectra of the gold-decorated MWCNTs before and after the deposition of the SAM monolayer. This technique provides useful information on the various chemical bonds present on the sensor structure.

#### Vapour sensing experimental setup

The sensors developed based on Au-MWCNTs and HDT/Au-MWCNTs were tested for the detection of aromatic VOCs (toluene and benzene). The vapours were generated by a dilution bench consisting of a chemical vaporisation cell for solvents and two flowmeters to generate reproducible concentrations of the different vapours tested. These were coupled to a sensor cell (35 cm^3^ volume) which can support up to six sensors at a time. The sensor resistance was measured using an Agilent HP 34972A multimeter at a fixed operating frequency of 1 kHz. Once a stable electrical resistance was achieved in the presence of the carrier gas (pure dry air, purchased from Air Liquide), we injected the adequate concentration of the target VOC. The flow rate was set to 200 standard cubic centimetres per minute. All measurements were carried out at room temperature and the response of the sensors was defined as the normalized resistance variation, presented in Equation 1 [21–22]

[1] $$\frac{\Delta R}{R_{0}}\left(\%\right)=\left[\frac{R_{\text{g}}-R_{0}}{R_{0}}\right]*100\;,$$

where *R*_0_ and *R*_g_ are the resistance under the carrier gas and the aromatic VOC, respectively.

## Results and Discussion

### Morphological and compositional characterisation

#### Transmission electron microscopy characterisation

**Figure 2:**
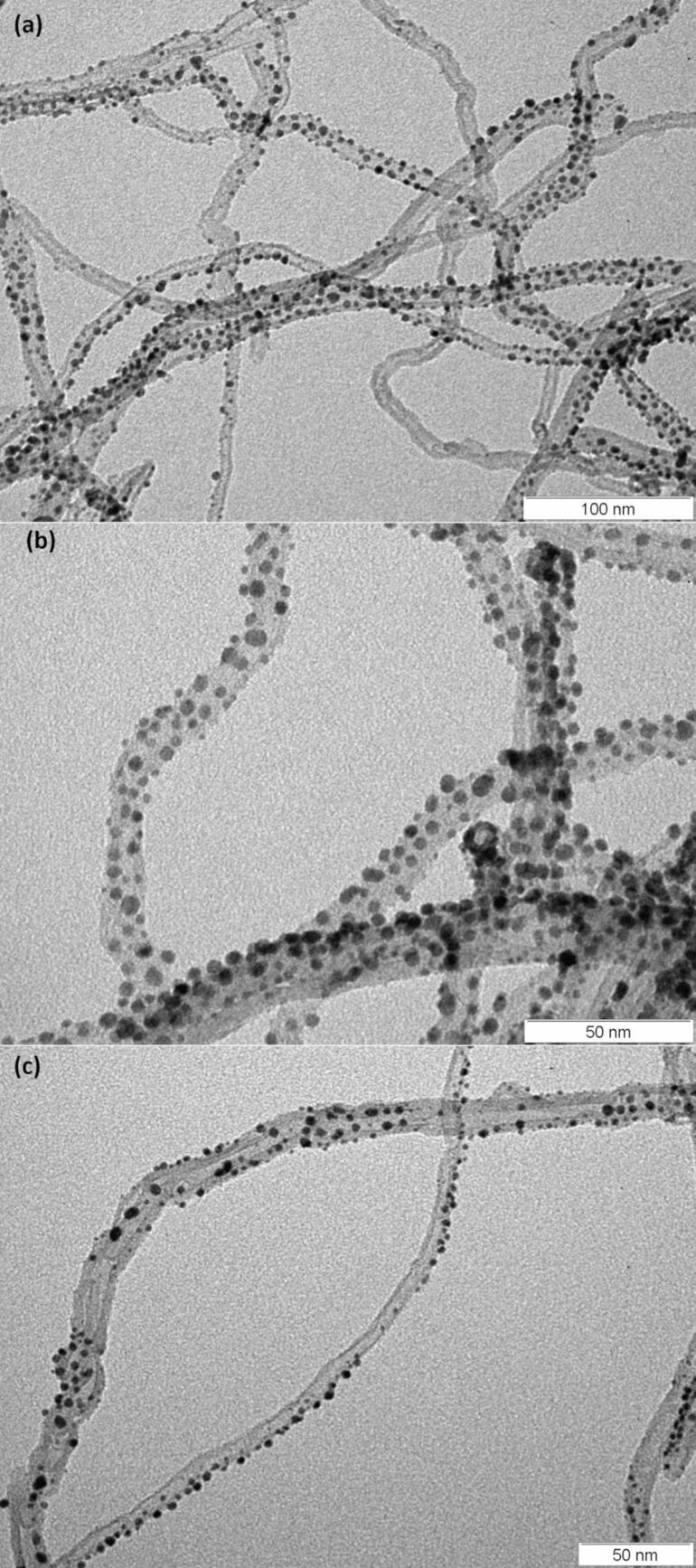
HRTEM image of MWCNTs decorated with Au nanoparticles at a magnification of (a) 300 K, (b) 600 K and (c) 400 K.

In order to carry out high-resolution transmission electron microscope characterisation, MWCNTs were deposited onto a silicon substrate. Then, a sputtering process was conducted to deposit the Au nanoparticles. Afterwards, Au-decorated MWCNTs were mechanically moved from the substrate onto a TEM Cu-grid for conducting TEM analysis. Figure 2 displays HRTEM images at a magnification of (a) 300 K, (b) 600 K and (c) 400 K. The HRTEM analysis indicates an average gold nanoparticle size of 2 nm.

We clearly see in Figure 2b a fairly homogeneous Au nanoparticle distribution. We must point out though, that as the sputtering deposition technique only applies to the most exposed surfaces, a shadowing effect is also observed. This effect is generally observed for porous substrates, where sputtering only reaches the outermost surface and the topmost areas of the pore openings efficiently [23–24]. In fact, in Figure 2a we see that CNTs with a higher number of Au nanoparticles were at the surface of the CNT mat while those showing fewer particles were deeper in the CNT film. Also, in Figure 2c, we can observe a nanotube that crosses the centre of the image where all of the nanoparticles are on one side only (the exposed side). Despite this fact, the Au nanoparticle decoration is considered sufficiently homogeneous for our application needs. The use of other techniques, resulting in a more efficient distribution, are believed to further enhance the overall sensor performance [25–26].

#### FTIR characterisation

**Figure 3:**
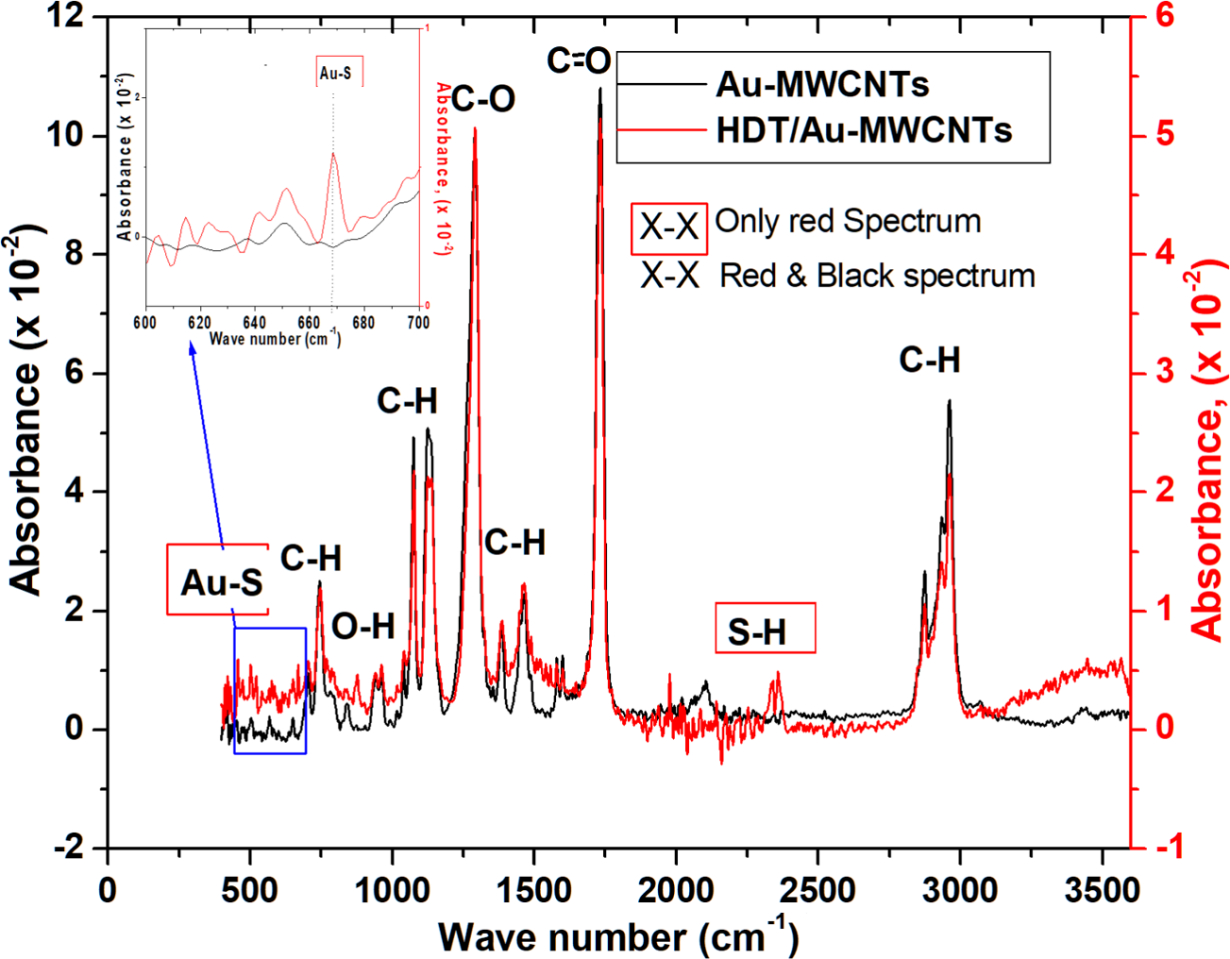
Infrared spectra of Au-MWCNT and SAM/Au-MWCNT layers.

In order to ensure that the HDT monolayers are formed and well immobilized on the Au-MWCNT layers, we used FTIR spectroscopy. Figure 3 shows the infrared spectra, in absorbance mode, of both Au-MWCNT and HDT/Au-MWCNT layers. When analysing and comparing the infrared spectra of Au-MWCNT and SAMs/Au-MWCNT layers, we found common bands and peaks associated with the carbonyl, carboxyl and hydroxyl groups attached to the MWCNT side walls during the oxygen plasma treatment. We also found common bands and peaks associated with the C–H bonds present in both layers. The corresponding bands are found at 2960, 1730, 1470, 1290 and 1076 cm^−1^, which can be assigned to C–H stretching, C=O stretching, C–H bending, C–O stretching and C–H anti-stretching vibration mode, respectively. The existence of the associated Au–S bond weak intensity peak positioned at 669 cm^−1^ proves the attachment of 1-hexadecanethiol on the Au-MWCNT sensor layer. The corresponding S–H stretch vibration mode band is found at 2360 cm^−1^ [27–30]. Moreover, other techniques were used for thiol monolayer characterisation. In previous work [31–32], different thiol chains were characterized by Raman spectroscopy, X-ray photoelectron spectroscopy (XPS) and contact angle measurements. In summary, the obtained FTIR results confirm the covalent functionalisation of Au-decorated MWCNTs with HDT.

### Sensing results

#### Au-MWCNT sensing of aromatic VOCs

**Figure 4:**
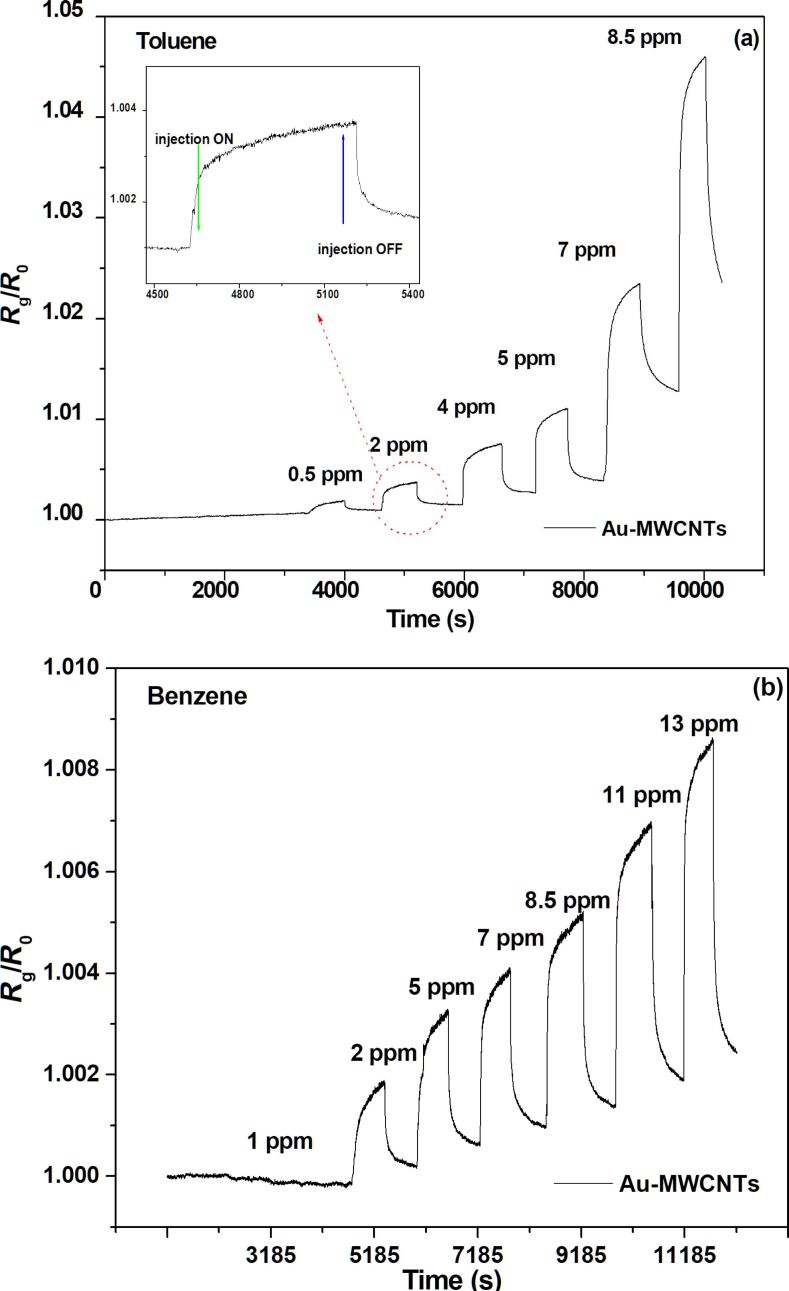
Au-MWCNT sensor response for different concentrations of the injected vapours of (a) toluene and (b) benzene.

Figure 4 shows the response of the Au-MWCNT sensor to benzene and toluene for various vapour concentrations injected at room temperature.

**Figure 5:**
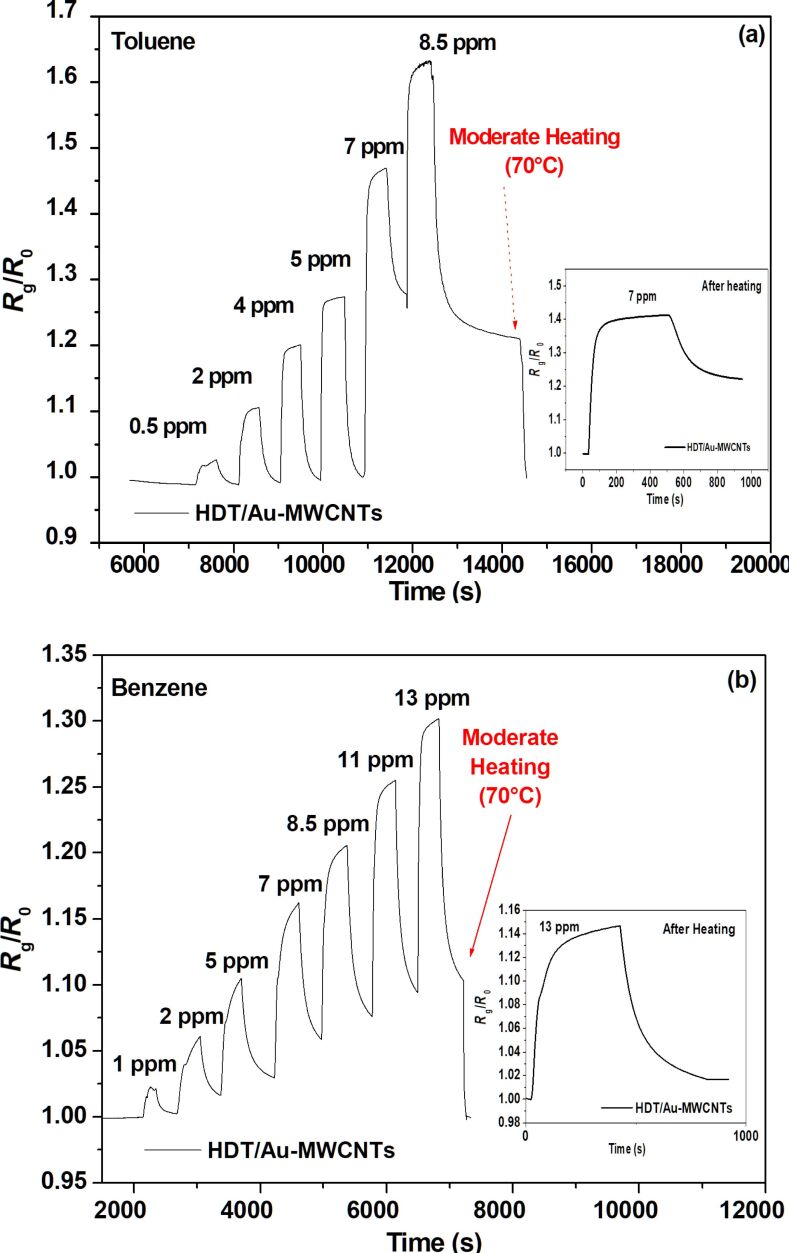
HDT/Au-MWCNT sensor responses for different concentrations of the injected vapours of (a) toluene and (b) benzene.

The results show an increasing resistance with increasing concentration of the injected vapours. The adsorption of these vapours on the Au-MWCNT films leads to a reduction of the global sensor electrical conductivity. The lowest concentrations measured were 2 ppm for benzene and 0.5 ppm for toluene. Physisorption is observed for toluene at concentrations below 7 ppm (below 5 ppm for benzene), indicating weak interaction forces between the vapour molecules and the Au-MWCNT active sensor layer and the signal returns to the baseline. For a higher concentration, a drift was observed in the baseline due to the higher interaction with toluene (and respectively benzene). This stronger interaction suggests slower baseline overlaps, which can be accelerated with short heating for few seconds at 70 °C (Figure 5).

#### HDT/Au-MWCNT sensing of aromatic VOCs

Figure 5 shows the response of the HDT/Au-MWCNT sensor to the varying benzene and toluene vapour concentrations injected at room temperature.

The detection results of the various injected vapours show a considerable decrease in the electrical conductivity of the HDT/Au-MWCNT sensor compared with the non-functionalised sensor. The same kinetics were also observed, where physisorption is visible for toluene below a concentration of 7 ppm (below 5 ppm for benzene). A short heating for a few seconds at 70 °C is sufficient for desaturating the sensor (desorbing the vapour molecules) and its return to the baseline. The figure insets represent the sensor response after heating at 70 °C for promoting baseline recovery. In addition, these measurements were performed eight months later after the initial measurements. They clearly show that the sensors respond after heating and after several months of operation/storage.

The weak interaction of the toluene molecule with the surface of both functionalised and non-functionalised sensors can be explained by the hydrophobic–hydrophobic interaction between the functional groups of the monolayer (CH_3_ groups) and the CH_3_ group of the toluene molecule. The considerable decrease in the conductivity is due to the oxidizing nature of the injected vapours. As the tested oxygen-treated and gold-decorated MWCNTs are p-type semiconductors, the adsorption of the oxidant vapour molecules leads to a transfer of the majority carriers of p-type semiconductors towards the adsorbed vapour molecules. This transfer results in a decrease of the majority carriers in the valence band of the decorated carbon nanotubes, and thus the decrease of the measured conductivity [33].

#### Sensor performance

**Figure 6:**
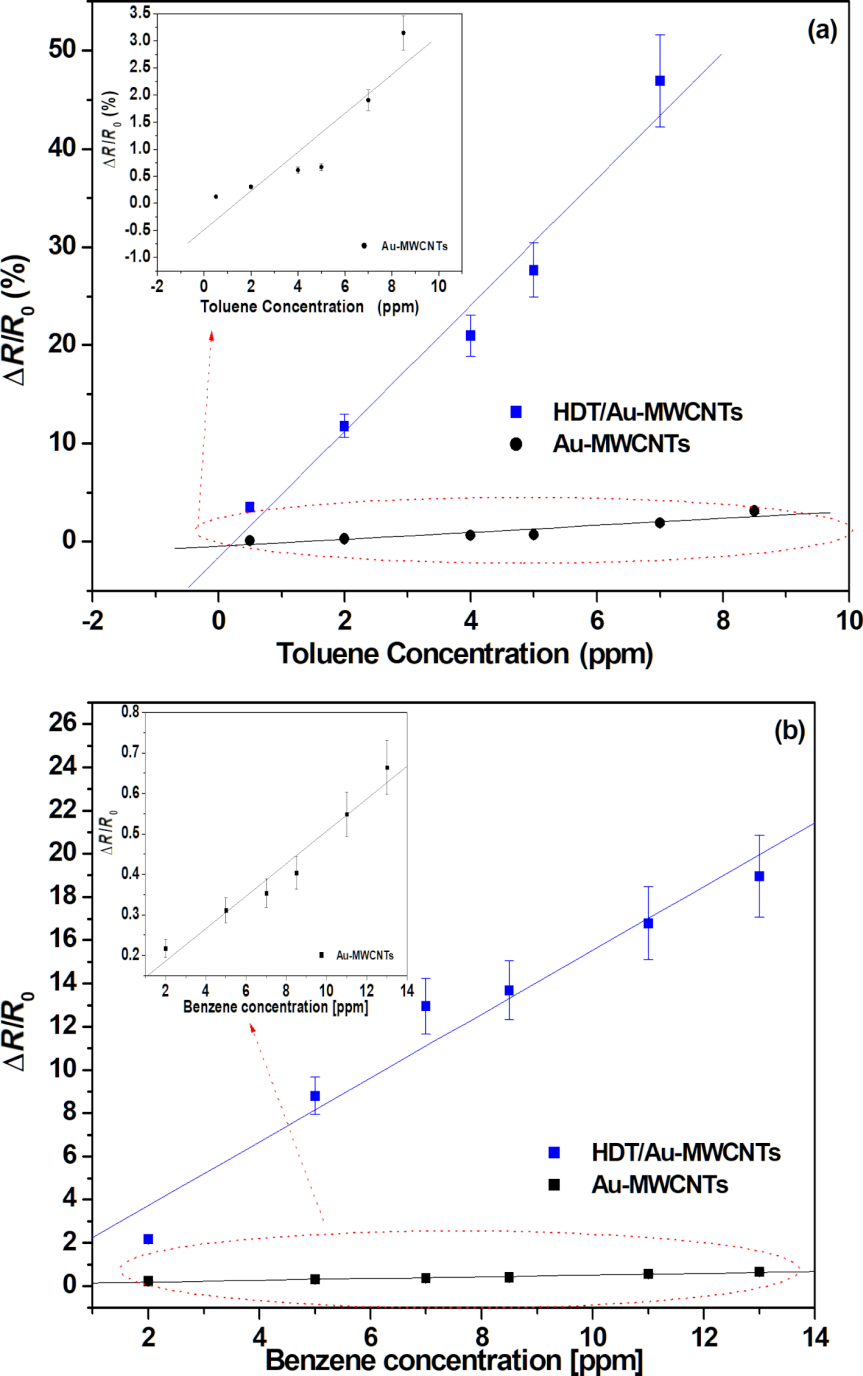
Calibration curves for Au-MWCNT and HDT/Au-MWCNT sensors for (a) toluene and (b) benzene aromatic VOC detection.

The calibration curves, expressing the normalized sensor resistance versus the vapour concentration for toluene and benzene, are presented in Figure 6a and Figure 6b, respectively, for both tested sensors. The associated sensitivities are displayed in Table 1.

**Table 1:** Au-MWCNT and HDT/Au-MWCNT sensor sensitivity for aromatic and nonaromatic vapours.

Sensitivity (10^−2^ %·ppm^−1^)	Toluene	Benzene	Methanol	Acetone

Au-MWCNT	35.8	4.8	3.15	0.84
HDT/Au-MWCNT	642.17	147	78.91	20.48

**Figure 7:**
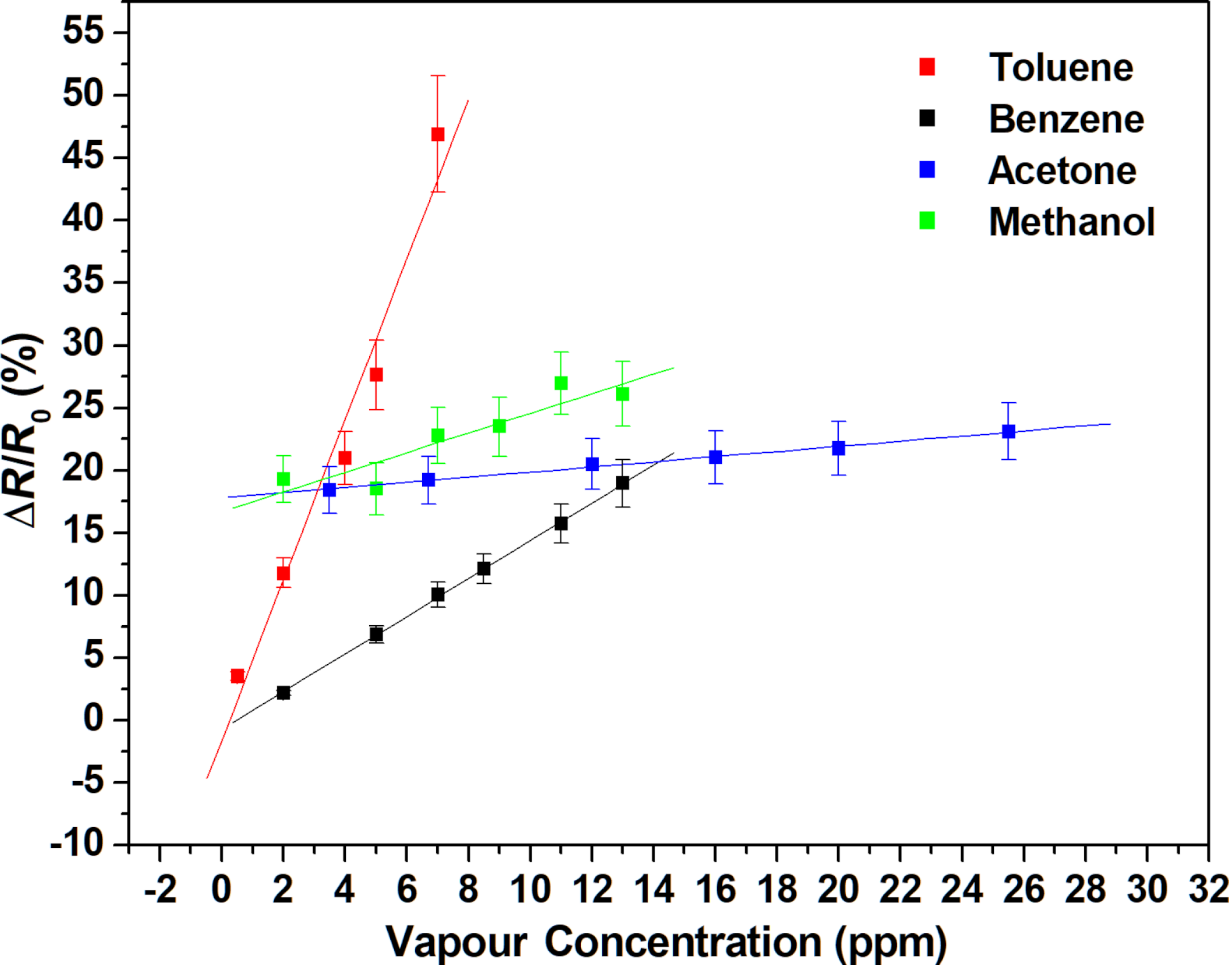
Calibration curves for the HDT/Au-MWCNT sensor for methanol and acetone nonaromatic VOC detection.

A dramatic increase in the HDT functionalised sensor sensitivity is observed for both vapours. The increase was from 4.79 × 10^−2^ %·ppm^−1^ to 147 × 10^−2^ %·ppm^−1^ for benzene and from 35.82 × 10^−2^ %·ppm^−1^ to 642.17 × 10^−2^ %·ppm^−1^ for toluene. It is noteworthy that the CH_3_ group in the HDT molecule seems to have a high affinity towards aromatic vapours, in contrast with the results presented in a previous work, where a 16-mercaptohexadecanoic acid (MHDA) showed no response to aromatic vapours [22]. As presented in Table 1, the vapours tested are nonpolar in nature and therefore have a high affinity with the hydrophobic CH_3_ group of the HDT. On the contrary, MHDA molecules are carboxylic acid terminated thiols, which are hydrophilic, and are unlikely to interact with the nonpolar vapours. In order to prove the improved selectivity of the developed sensor toward benzene and toluene vapours, we tested the sensor response to methanol and acetone vapours, which are polar solvents. The sensor response to these nonaromatic VOC vapours are summarized in Supporting Information File 1 (Figures S1 and S2). The associated calibration curves and compared sensitivities are presented in Figure 7 and Table 1, respectively. The sensitivity of the HDT sensors with aromatic solvents is larger in comparison with methanol and acetone. This can be explained by the fact that the assembled CH_3_ group acts as a Lewis acid and the latter one (benzene and toluene) as a Lewis base [34]. The sensitivity for toluene is higher than for benzene due to the difference in their dipole moment (toluene: 0.375; benzene: 0).

The effect of ambient moisture on the sensor response was studied in a previous work [32]. The response to vapours remains basically unchanged for sensors employing hydrophobic thiols due to their hydrophobicity [31]. Such long-chain alkanethiol functionalised and decorated MWCNTs showed a high contact angle with water, which remains stable after a one month stay in aqueous media [31].

**Figure 8:**
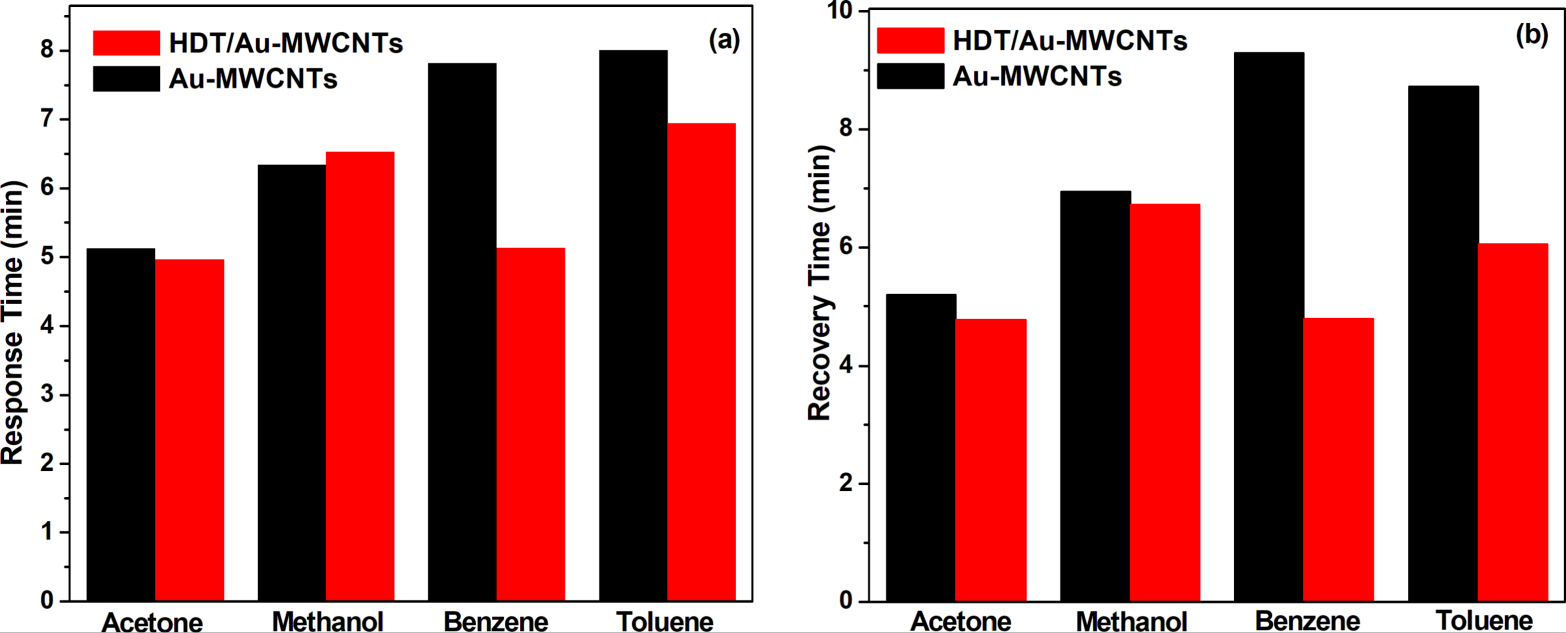
(a) Response and (b) recovery times of Au-MWCNT and HDT/Au-MWCNT sensors towards tested vapours.

Figure 8 presents the response and recovery times of Au-MWCNT and HDT/Au-MWCNT sensors toward aromatic and nonaromatic vapours. The results show faster response and recovery times for the HDT-functionalised Au-MWCNT sensor with benzene and toluene vapours.

## Conclusion

In this work, we studied the detection of aromatic vapours in ppm range with a sensor composed of HDT-functionalised gold-decorated multiwall carbon nanotubes. The studied self-assembled monolayer, with its CH_3_ functional group, increased the sensor sensitivity (up to 17 times) and selectivity. It was also shown to improve the sensor response dynamics. These results combined with previous results [22,32] could be interesting for the development of functionalised multisensor arrays combined with an artificial intelligence algorithm for selectivity enhancement.

## Supporting Information

File 1 Details on the HDT/Au-MWCNT sensor fabrication process and its response to methanol and acetone vapours.
