# Supplementary material for: Multiwalled carbon nanotube based aromatic volatile organic compound sensor: sensitivity enhancement through 1-hexadecanethiol functionalisation
Source: Beilstein J Nanotechnol. 2019 Dec 4;10:2364–73. doi: 10.3762/bjnano.10.227 (PMC6902881; doi:10.3762/bjnano.10.227)
Supplement: File 1 — Details on the HDT/Au-MWCNT sensor fabrication process and its response to methanol and acetone vapours. [file Beilstein_J_Nanotechnol-10-2364-s001.pdf]

BEILSTEIN JOURNAL OF NANOTECHNOLOGY

Supporting Information

for

Multiwalled carbon nanotube based aromatic volatile organic compound sensor: sensitivity enhancement through 1-hexadecanethiol functionalisation

Nadra Bohli, Meryem Belkilani, Juan Casanova-Chafer, Eduard Llobet and Adnane Abdelghani

Beilstein J. Nanotechnol. **2019,** *10,* 2364–2373. doi:10.3762/bjnano.10.227

Details on the HDT/Au-MWCNT sensor fabrication process and its response to methanol and acetone vapours

License and Terms: This is a supporting information file under the terms of the Creative Commons Attribution License (http://creativecommons.org/licenses/by/4.0). Please note that the reuse, redistribution and reproduction in particular requires that the authors and source are credited.
The license is subject to the *Beilstein Journal of Nanotechnology* terms and conditions: (https://www.beilstein-journals.org/bjnano)

Detailed description of the MWCNT oxygen plasma treatment

MWCNTs were placed in an ATC Orion-8-HV multi-target sputtering machine chamber under a controlled flow of oxygen and argon (AJA International, Inc., USA). The sputtering machine parameters were set at a pressure of 0.1 Torr and a power of 15 W for 1 min. The plasma was coupled inductively at a frequency of 13.56 MHz. The resulting functional oxygen species created on the carbon nanotube sidewalls were carboxyl, carbonyl and hydroxyl groups.

Sensor response to methanol and acetone vapors

The figures related to HDT/Au-MWCNTs sensor response to methanol and acetone vapours are presented respectively in Figures S1 and S2.

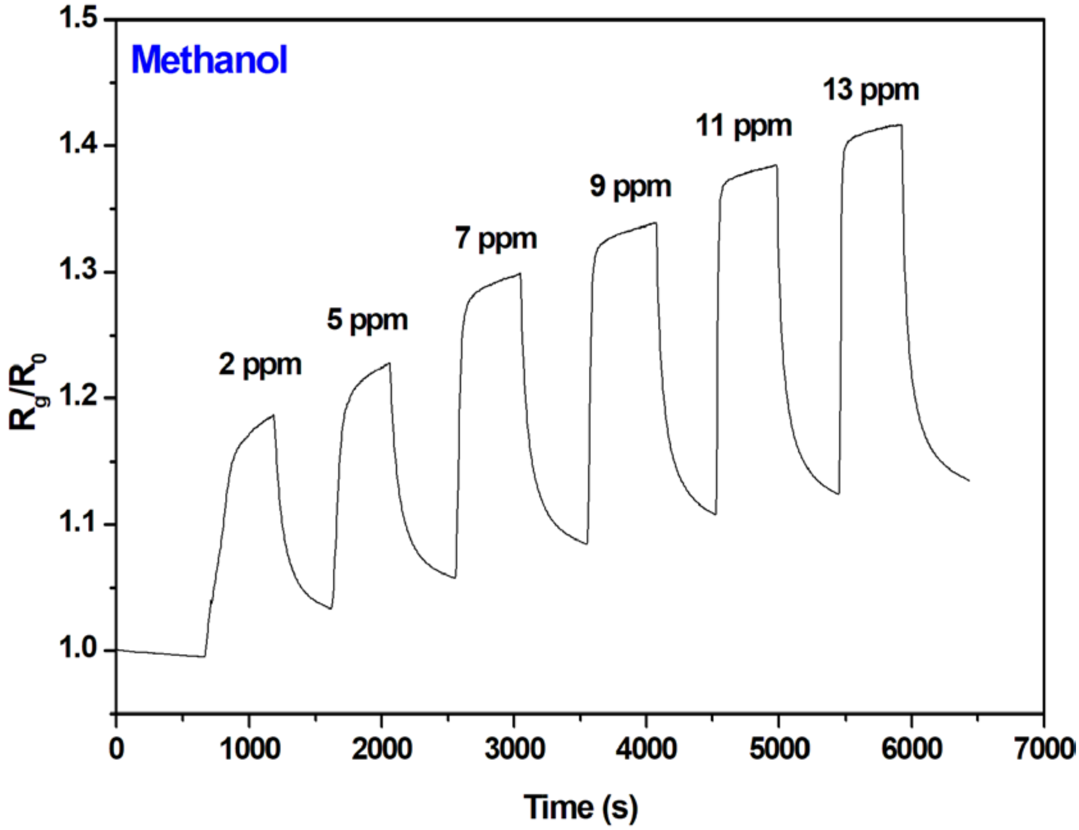

Figure S1: HDT/Au-MWCNT sensor response for methanol nonaromatic VOC detection.

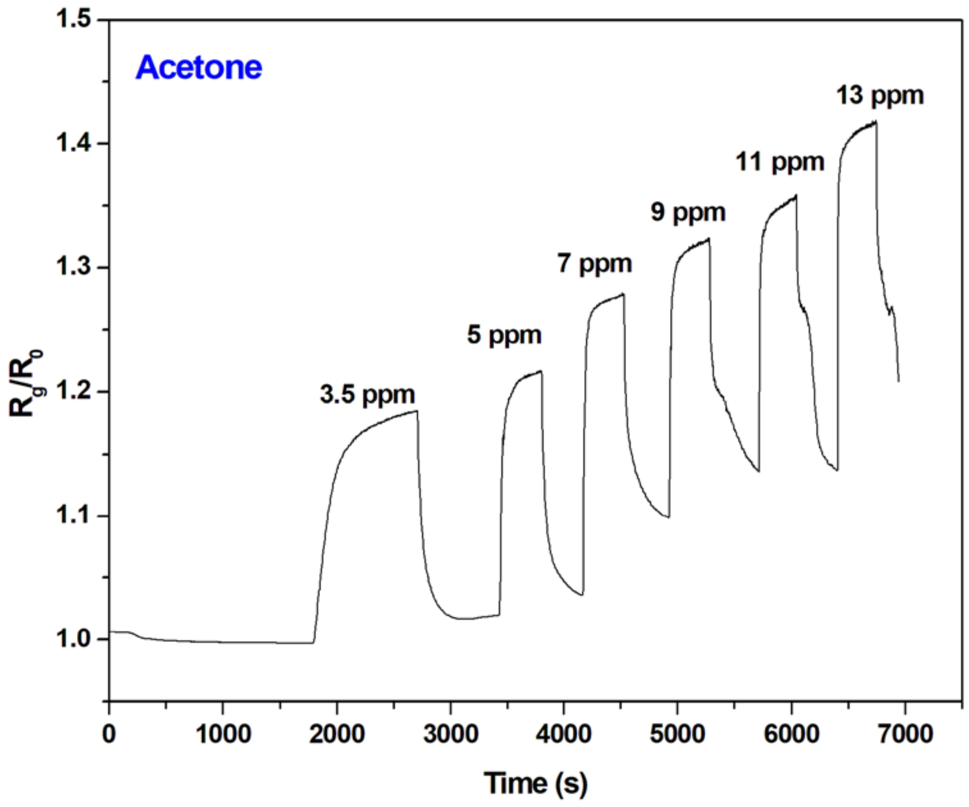

Figure S2: HDT/Au-MWCNT sensor for acetone nonaromatic VOC detection.
